# m^6^A RNA modification and its writer/reader VIRMA/YTHDF3 in testicular germ cell tumors: a role in seminoma phenotype maintenance

**DOI:** 10.1186/s12967-019-1837-z

**Published:** 2019-03-12

**Authors:** João Lobo, Ana Laura Costa, Mariana Cantante, Rita Guimarães, Paula Lopes, Luís Antunes, Isaac Braga, Jorge Oliveira, Mattia Pelizzola, Rui Henrique, Carmen Jerónimo

**Affiliations:** 1 Cancer Biology and Epigenetics Group (CBEG), IPO Porto Research Center (CI-IPOP), Portuguese Oncology Institute of Porto (IPO Porto) & Porto Comprehensive Cancer Center (P.CCC), R. Dr. António Bernardino de Almeida, 4200-072 Porto, Portugal; 2 Department of Pathology, Portuguese Oncology Institute of Porto (IPOP), R. Dr. António Bernardino de Almeida, 4200-072 Porto, Portugal; 3 Department of Pathology and Molecular Immunology, Institute of Biomedical Sciences Abel Salazar, University of Porto (ICBAS-UP), Rua Jorge Viterbo Ferreira 228, 4050-513 Porto, Portugal; 4 Department of Epidemiology, Portuguese Oncology Institute of Porto (IPOP), R. Dr. António Bernardino de Almeida, 4200-072 Porto, Portugal; 5 Department of Urology, Portuguese Oncology Institute of Porto (IPOP), R. Dr. António Bernardino de Almeida, 4200-072 Porto, Portugal; 6 Center for Genomic Science of IIT@SEMM, Fondazione Istituto Italiano di Tecnologia (IIT), 20139 Milan, Italy

**Keywords:** Epitranscriptomics, M^6^A, RNA, Testicular germ-cell tumors, VIRMA, YTHDF3

## Abstract

**Background:**

Covalent RNA modifications, such as N-6-methyladenosine (m^6^A), have been associated with various biological processes, but their role in cancer remains largely unexplored. m^6^A dynamics depends on specific enzymes whose deregulation may also impact in tumorigenesis. Herein, we assessed the differential abundance of m^6^A, its writer VIRMA and its reader YTHDF3, in testicular germ cell tumors (TGCTs), looking for clinicopathological correlates.

**Methods:**

In silico analysis of TCGA data disclosed altered expression of VIRMA (52%) and YTHDF3 (48%), prompting subsequent validation. Formalin-fixed paraffin-embedded tissues from 122 TGCTs (2005–2016) were selected. RNA extraction, cDNA synthesis and real-time qPCR (Taqman assays) for VIRMA and YTHDF3 were performed, as well as immunohistochemistry for VIRMA, YTHDF3 and m^6^A, for staining intensity assessment. Associations between categorical variables were assessed using Chi square and Fisher’s exact test. Distribution of continuous variables between groups was compared using the nonparametric Mann–Whitney and Kruskal–Wallis tests. Biomarker performance was assessed through receiver operating characteristics (ROC) curve construction and a cut-off was established by Youden’s index method. Statistical significance was set at p < 0.05.

**Results:**

In our cohort, VIRMA and YTHDF3 mRNA expression levels differed among TGCT subtypes, with Seminomas (SEs) depicting higher levels than Non-Seminomatous tumors (NSTs) (p < 0.01 for both). A positive correlation was found between VIRMA and YTHDF3 expression levels. VIRMA discriminated SEs from NSTs with AUC = 0.85 (Sensitivity 77.3%, Specificity 81.1%, PPV 71.6%, NPV 85.3%, Accuracy 79.7%). Immunohistochemistry paralleled transcript findings, as patients with strong m^6^A immunostaining intensity depicted significantly higher VIRMA mRNA expression levels and stronger VIRMA immunoexpression intensity (p < 0.001 and p < 0.01, respectively).

**Conclusion:**

Abundance of m^6^A and expression of *VIRMA*/*YTHDF3* were different among TGCT subtypes, with higher levels in SEs, suggesting a contribution to SE phenotype maintenance. VIRMA and YTHDF3 might cooperate in m^6^A establishment in TGCTs, and their transcript levels accurately discriminate between SEs and NSTs, constituting novel candidate biomarkers for patient management.

**Electronic supplementary material:**

The online version of this article (10.1186/s12967-019-1837-z) contains supplementary material, which is available to authorized users.

## Background

Testicular cancer is the most common neoplasia among Caucasian men aged 15–44 years, with rising incidence due to widespread adoption of Western lifestyle, with 65,827 new cases expected worldwide in 2030 [1–3]. More than 95% of cases are derived from germ cells—testicular germ cell tumors (TGCTs)—and the vast majority of these correspond to germ-cell neoplasia in situ (GCNIS)-related tumors, according to the most recent World Health Organization (WHO) classification [4]. Furthermore, this category comprises two major subtypes—seminomas (SEs) and non-seminomatous tumors (NSTs)—and discrimination between them is of paramount clinical importance, entailing different prognosis and treatment algorithms [5, 6].

TGCTs are fascinating tumors, in part because they are truly developmental cancers [7]. Heterogeneity is the hallmark of GCNIS-related TGCTs, reflecting this complex tumor model, although they share a common cytogenetic background, i.e., isochromosome 12p [4, 8]. Thus, biological, morphological and clinical heterogeneity might also be related with dissimilar epigenetic backgrounds, which might be surveyed using novel biomarkers. Indeed, there is an increasing need for reliable and clinically validated TGCT biomarkers that might improve diagnosis, subtype discrimination, prognostication and patient monitoring, overcoming the limitations of classical serum markers currently employed in the clinical setting [9–14].

Recently, post-translational RNA modifications (so-called “Epitranscriptomics”) have emerged as fundamental modulators of many biological and disease processes [15, 16]. The most abundant of these modifications, N6-methyladenosine (m^6^A), is dynamically regulated by a variety of m^6^A-related proteins, organized as writers (which catalyze the methyl code), erasers (which delete the methyl code) and readers (m^6^A-binding proteins that target RNAs to their final destiny) [17–22]. The amount of m^6^A modification in messenger RNAs (mRNAs) has been implicated in diverse biological mechanisms and related diseases, such as immune response, metabolism, gametogenesis, embryogenesis, neurodevelopment and cancer [23–27].

Deregulation of m^6^A-related proteins has been shown to impact tumorigenesis and progression of several neoplasms, including breast, lung, liver and colorectal carcinomas, leukemias and glioblastoma, among others [24, 28–31]. Recently, we have thoroughly reviewed the available literature concerning these players in all tumor models [32]. There have been several studies, focusing both on the writer METTL3 [33] and other components of the methylation complex (such as METTL14 [34]), on erasers (such as ALKBH5 [35]) and readers (such as YTHDF1 [36]), seeking for clinicopathological correlates or further upstream or downstream (de)regulation mechanisms in cancer. While most studies seem to imply that overexpression of writers associates with poor prognostic features, many exceptions were depicted. However, no studies have focused on VIRMA and YTHDF3 as a writer/reader pair, which is surprising since our in silico analysis of publicly available databases pointed out these players as being preferably deregulated in urological cancers, including prostate, kidney and bladder cancer and also in TGCTs.

Moreover, and to the best of our knowledge, no studies on TGCTs have been reported, despite the importance of m^6^A modification in germ cell differentiation [37]. Given this link between m^6^A modification and embryogenesis and spermatogenesis, and since TGCTs are developmental-related, we hypothesize that alterations in m^6^A amount may also have a role in these tumors, with possible differences among more undifferentiated forms such as SE and more differentiated subtypes such as Teratoma (TE).

## Methods

Given this rationale, we set out to assess the differential expression of m^6^A writer VIRMA and reader YTHDF3, in a cohort of TGCT patients, comparing with m^6^A abundance and establishing associations with clinicopathological data, looking for potential biological and clinical relevance of these findings.

### Patients and tissue sample collection

All patients presenting with TGCTs at the Portuguese Oncology Institute of Porto between 2005 and 2016 were retrospectively queried using the Department of Pathology’s database. Thus, a cohort of 122 consecutive GCNIS-related TGCT patients with material available for analysis was selected for this study. All patients were operated and subsequently treated at our Institution by the same multidisciplinary team. This study was approved by the ethics committee of Portuguese Oncology Institute of Porto (Comissão de Ética para a Saúde—CES-IPO-1-2018).

Clinical files and pathology reports were reviewed. All histological slides (of both primary tumors and matching metastatic specimens) were reviewed and tumors were reclassified in light of the most recent *2016 World Health Organization (WHO) Classification of Tumours of the Urinary System and Male Genital Organs* [4]. Staging was performed according to the 8^th^ edition of the *American Joint Committee on Cancer* (AJCC) staging manual. Patients presenting with metastases at diagnosis were further properly classified according to the *International Germ Cell Cancer Collaborative Group* (IGCCCG) prognostic system [38, 39]. Follow-up was last updated on November 30, 2017.

All tumor samples corresponded to formalin-fixed paraffin-embedded (FFPE) orchiectomy specimens (prior to any systemic treatment) and matched metastatic specimens. Representative blocks (with > 80% tumor cellularity) were selected and individual tumor areas were thoroughly macro-dissected (eliminating areas of necrosis or exuberant inflammation), considering each tumor subtype/component in mixed germ cell tumors (MGCTs) as an independent sample. Ten micrometer sections were obtained for subsequent RNA extraction and 5 μm sections for immunohistochemistry (IHC) assays.

### RNA extraction, cDNA synthesis and RT-qPCR

Total RNA was extracted using FFPE RNA/DNA Purification Plus Kit (Cat. 54300, Norgen), according to manufacturer instructions. RNA quantification and purity were assessed in NanoDrop™ Lite Spectophotometer (Cat. ND-LITE, Thermo Scientific™). cDNA synthesis (1000 ng) was accomplished by reverse transcription using RevertAid™ RT Reverse Transcription Kit (Cat. K1691, Thermo Scientific™). The reaction was performed in MyCycler™ Thermal Cycler System (Cat. 1709703, Bio-Rad) using the following conditions: 5 min at 25 °C, 60 min at 42 °C and 5 min at 70 °C. Samples were then stored at − 20 °C.

Real-time quantitative Polymerase Chain Reaction (RT-qPCR) was performed in LightCycler^®^ 480 multiwell plate system (Product no. 05015243001, Roche), according to the recommended protocol. TaqMan™ gene expression assays for VIRMA (assay ID Hs00936421, Thermo Fisher Scientific, Life Technologies^®^) and YTHDF3 (assay ID Hs00405590, Thermo Fisher Scientific, Life Technologies^®^) were used. For normalization of the assay (guaranteeing stable levels among all tumor samples) two normalizing TaqMan™ gene expression assays (beta-glucoronidase—GUSB—assay ID Hs99999908, Applied biosystems^®^; and 18S ribosomal RNA—18S rRNA, assay ID Hs99999901, Applied biosystems^®^) were used as internal controls. Mean concentration of both normalizing genes was calculated and relative expression of targets tested in each sample were obtained using the formula: ${\text{Relative Expression}}\, = \,\frac{{\varvec{Target} \ \varvec{gene} \ \varvec{mean} \ \varvec{quantity}}}{{(\varvec{\beta GUS} + 18\varvec{S}) \ \varvec{mean} \ \varvec{quantity}}}$. The ratio obtained was then multiplied by 1000 for easier tabulation. Serial dilutions of cDNA obtained from Human Reference Total RNA (Cat. 750500, Agilent Technologies^®^) were used to compute standard curves for each plate. All experiments were run in triplicate and two negative controls were used in each plate.

### Immunohistochemistry

Antigenic recovery was performed with EDTA buffer in water bath (40 min) and endogenous peroxidase activity was blocked by 0.6% hydrogen peroxide. Nonspecific reactions were blocked with normal horse serum (dilution 1:50). Slides were incubated overnight with the following primary antibodies: anti-VIRMA rabbit polyclonal (Cat. PA5-56772, RRID AB_2643047, dilution 1:100; Thermo Fisher Scientific^®^); anti-YTHDF3 rabbit polyclonal (Product code ab103328, dilution 1:100, Abcam^®^); and anti-m^6^A rabbit monoclonal (Product code ab190886, dilution 1:750, Abcam^®^). Both post-primary antibody and polymer were incubated for 30 min at room temperature (Novolink™ Polymer Detection System—Novocastra, Product No. RE7150-K). Diaminobenzidine was used as chromogen and hematoxylin as counterstain. Urothelial carcinoma, breast carcinoma and normal brain tissue were used as positive controls for VIRMA, YTHDF3 and m^6^A, respectively. Negative control consisted on omission of primary antibodies. However, when the protocols were developed, negative external controls were used. Immunoexpression intensity was estimated separately for each TGCT component (including different components among MGCTs), considering staining as “weak”, “moderate” or “intense”; the percentage of stained cells was also assessed, but the staining was rather homogeneous among tumor samples, with all cells showing “positive” staining.

### Statistical analysis

Data was tabulated using Microsoft Excel 2016 and analyzed using IBM SPSS Statistics version 24 and GraphPad Prism 6. Percentages were calculated based on the number of cases with available data. Associations between categorical variables were assessed using Chi square and Fisher’s exact test, and group proportions were compared with odds ratios (ORs). Distribution of continuous variables between groups was compared using the nonparametric Mann–Whitney test and Kruskal–Wallis test, as appropriate. Bonferroni’s correction and Dunn’s test were employed for adjusting p-values in multiple comparisons, as appropriate. Correlation between continuous variables was assessed with Spearman’s (r_s_) non-parametric correlation test and interpretation of strength of results according to the system proposed by Evans [40]. In patients diagnosed with MGCT, the highest expression levels among all tumor components was considered for evaluating associations with clinicopathological features. Biomarker performance was assessed through receiver operating characteristics (ROC) curve construction. ROC curves were constructed plotting sensitivity (true positive) against 1-specificity (false positive). A cut-off was established by Youden’s index method [41, 42]. In addition, area under the curve (AUC) and biomarker performance parameters, including sensitivity, specificity, positive predictive value (PPV), negative predictive value (NPV) and accuracy, were ascertained. A logistic regression model was built to assemble the expression levels of both biomarkers as a panel. A p value ≤ 0.05 was considered statistically significant.

## Results

### In silico analysis

In silico analysis of the publicly available *The Cancer Genome Atlas* (TCGA) database for TGCTs concerning m^6^A-related proteins (writers, erasers and readers) was carried out. For this purpose, the online resource *cBioPortal for Cancer Genomics* was used [43] with the user-defined entry gene set “*METTL3*, *METTL14*, *METTL4*, *METTL16*, *WTAP*, *VIRMA*, *RBM15*, *RBM15B*, *FTO*, *ALKBH5*, *YTHDF1*, *YTHDF2*, *YTHDF3*, *YTHDC1*, *YTHDC2*, *EIF3A*, *HNRNPC* and *HNRNPA2B1*″. The database includes 156 tumors samples [65 SEs (42%) and 91 NSTs (58%)] from 150 patients, with a median age at diagnosis of 31 years. Most patients were stage I (79.4%) and considering patients with metastatic disease, most belonged to the good prognosis group (74.4%).

**Fig. 1:**
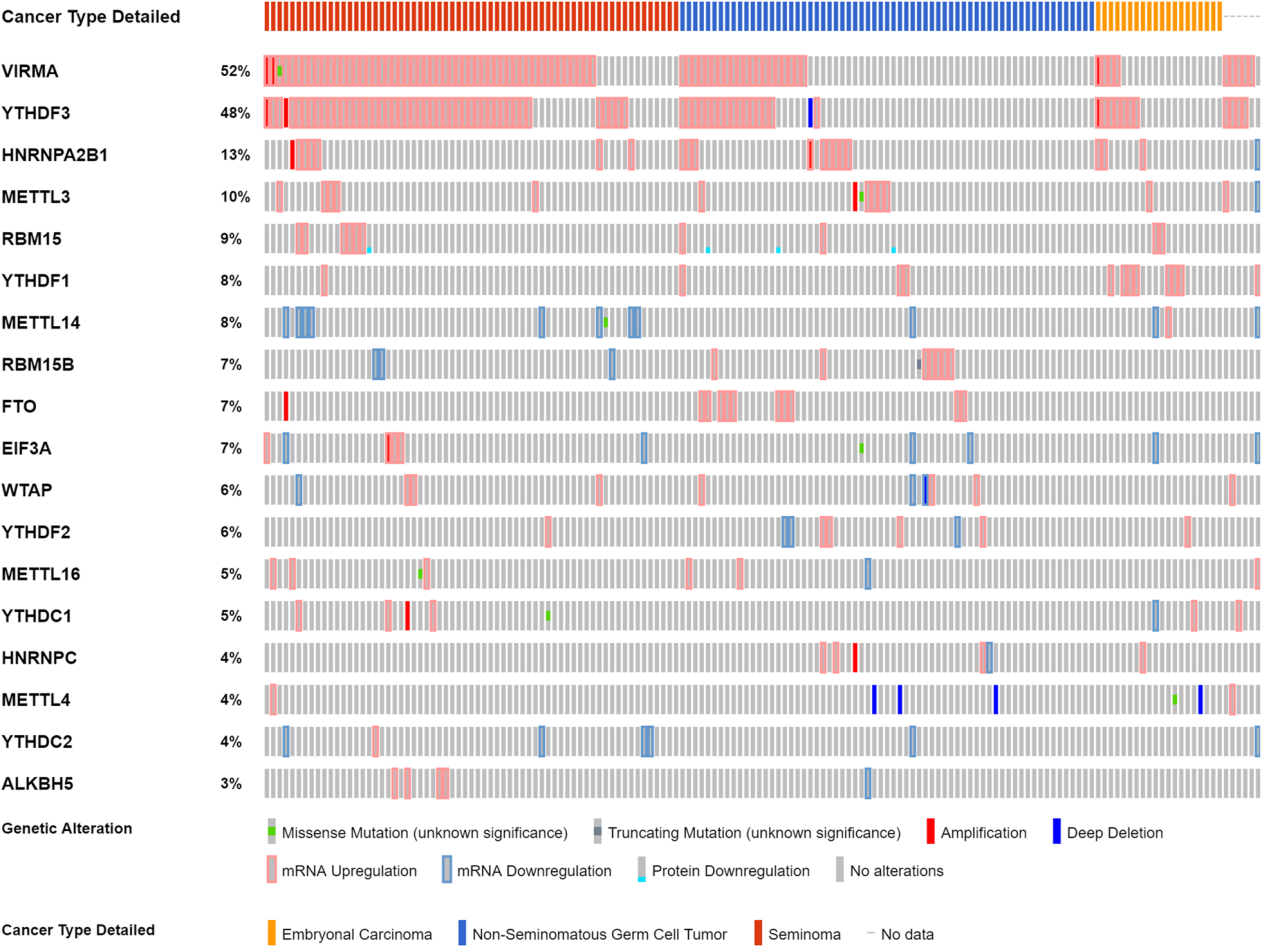
In silico analysis: frequency of alterations in queried genes in TCGA database. Notice the frequency of alterations in VIRMA and YTHDF3 compared to other queried genes. Also, note the frequent co-existence of alterations in this pair of genes, and the high proportion of seminomas showing deregulation of such genes

TCGA dataset analysis revealed that *KIAA1429*/*VIRMA* (a player belonging to the m^6^A writer complex) and *YTHDF3* (m^6^A reader) were the two most commonly altered m^6^A-related genes in TGCTs (52% and 48% of the samples, respectively). Most alterations consisted of transcript upregulation, with no mutations found for *YTHDF3* and only one depicted for *VIRMA* (Fig. 1). Importantly, the analysis of TCGA data depicted a strong correlation between mRNA expression levels of both these m^6^A regulators (r_s_ = 0.77). Based on the results of this in silico analysis, VIRMA and YTHDF3 were selected for further validation in a patient tissue cohort.

In the same dataset, transcript levels of both genes were significantly higher in SE and in stage I disease (p < 0.001 for both), with VIRMA achieving an AUC of 0.83 in the discrimination between SEs and NSTs, using ROC curve analysis (Additional file 1: Figure S1).

### IPO Porto’s cohort: characterization and validation

A total of 122 GCNIS-related TGCT patients were included in this study, comprising 56 (46%) with pure SE and 66 (54%) with NSTs, which included 58 MGCTs. Considering each tumor component as an independent sample (in a similar approach already used by us [44, 45]), we obtained a series of 75 SE (56 pure and 19 as MGCT component), 39 embryonal carcinomas (ECs) (5 pure and 34 as MGCT component), 35 postpubertal-type yolk sac tumors (YSTs) (all as MGCT components), 12 choriocarcinomas (CHs) (all as MGCT components) and 36 postpubertal-type TEs (3 pure and 33 as MGCT components). Most patients were stage I (78/122, 63.9%) and among patients with metastatic disease, most belonged to the good prognosis group (31/44, 70.5%). Detailed cohort description is depicted in Table 1.

**Table 1:** Clinicopathological characteristics of the testicular germ cell tumor cohort

Clinicopathological features	TGCT patients (n = 122)
Median age, years (IQR)	28 (24–36)
Histological type	
Seminoma	56/122 (45.9%)
Embryonal carcinoma	5/122 (4.1%)
Postpubertal-type yolk sac tumor	0/122 (0%)
Choriocarcinoma	0/122 (0%)
Postpubertal-type teratoma	3/122 (2.5%)
Mixed tumor	58/122 (47.5%)
Pathological stage	
I	78/122 (63.9%)
II	23/122 (18.9%)
III	21/122 (17.2%)
IGCCCG grouping (for metastatic disease)	
Good	31/44 (70.5%)
Intermediate	6/44 (13.6%)
Poor	7/44 (15.9%)

Clinicopathological features	Primary TGCT tumor samples (n = 197)
Histological type	
Seminoma	75/197 (38.0%)
Embryonal Carcinoma	39/197 (19.8%)
Postpubertal-type yolk sac tumor	35/197 (17.8%)
Choriocarcinoma	12/197 (6.1%)
Postpubertal-type teratoma	36/197 (18.3%)

Clinicopathological features	Metastatic TGCT tumor samples (n = 19)
Histological type	
Seminoma	1/19 (5.3%)
Embryonal carcinoma	5/19 (26.3%)
Postpubertal-type yolk sac tumor	3/19 (15.8%)
Choriocarcinoma	1/19 (5.3%)
Postpubertal-type teratoma	9/19 (47.3%)
Type of metastasis	
Lymph node	12/19 (63.2%)
Visceral	7/19 (36.8%)

*IGCCCG* International Germ Cell Cancer Collaborative Group, *IQR* interquartile range, *TGCT* testicular germ cell tumors

### VIRMA and YTHDF3 mRNA expression levels in IPO Porto’s cohort

#### SE vs. NST tissue samples

**Fig. 2:**
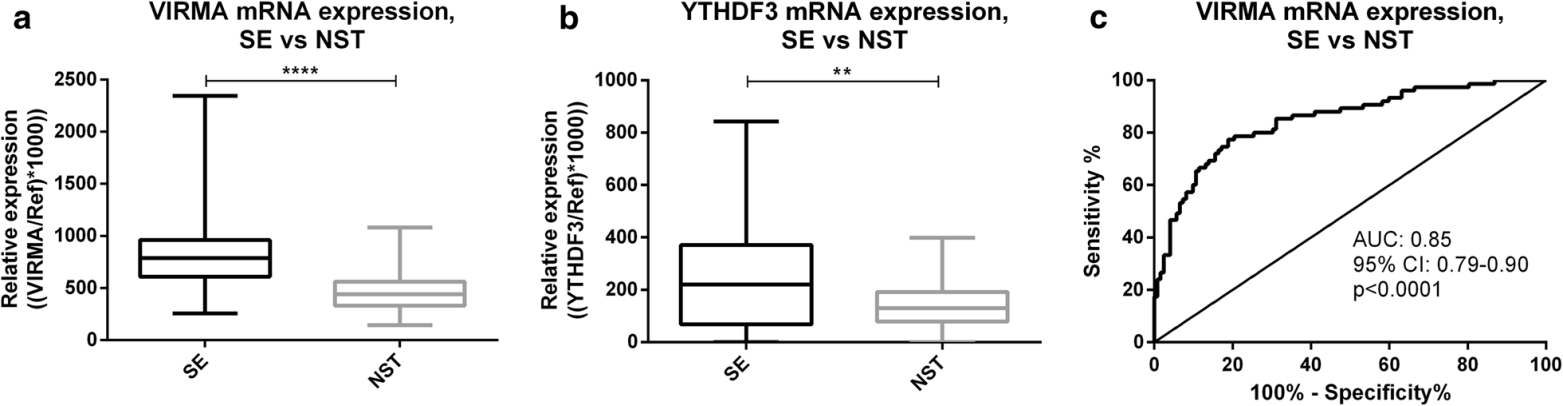
Transcript levels of VIRMA and YTHDF3 among seminoma and non-seminomatous tumor samples. **a** VIRMA mRNA expression in seminomas vs. non-seminomatous tumors; **b** YTHDF3 mRNA expression in seminomas vs. Non-seminomatous tumors; **c** ROC curve for discrimination among seminomas and non-seminomatous tumors based on VIRMA mRNA expression levels. *SE* Seminoma, *NST* non-seminomatous tumor, *AUC* area under the curve, *Ref* reference genes GUSB and 18S, *CI* confidence interval

*VIRMA* and *YTHDF3* mRNA levels were significantly higher in SEs compared to NSTs (p < 0.001 and p = 0.0014, respectively) (Fig. 2a, b). Moreover, *VIRMA* transcript levels discriminated SEs from NSTs with 77.3% sensitivity, 81.1% specificity, 72.0% PPV, 86.1% NPV and 80.2% accuracy, corresponding to an AUC of 0.85 in ROC curve analysis (Fig. 2c). Nonetheless, *YTHDF3* discriminative power was rather modest: 53.3% sensitivity, 81.1% specificity, 63.5% PPV, 73.9% NPV, 70.6% accuracy and an AUC of 0.64.

Combining expression levels of both genes in a logistic regression model, *YTHDF3* did not significantly increment the performance of *VIRMA* transcript. Indeed, a similar AUC value was depicted (AUC = 0.84). Nevertheless, considering the VIRMA/YTHDF3 panel “positive” if at least one of the genes was expressed above the empirical cutoff value, high sensitivity (82.7) and NPV (87.1%) were achieved (Table 2).

**Table 2:** Performance parameters for discriminating among Seminomas and Non-Seminomatous Tumors

Gene/panel	AUC (95% CI)	Sensitivity (%)	Specificity (%)	PPV (%)	NPV (%)	Accuracy (%)
VIRMA	0.85 (0.79–0.90)	77.3	81.1	72.0	86.1	80.2
YTHDF3	0.64 (0.55–0.72)	53.3	81.1	63.5	73.9	70.6
VIRMA/YTHDF3 (≥ 1 above cutoff)	–	82.7	72.1	64.6	87.1	76.1
VIRMA/YTHDF3 (both above cutoff)	–	48.0	90.2	75.0	73.8	74.1

*AUC* area under the curve, *CI* confidence interval, *PPV* positive predictive value; *NPV* negative predictive value

#### Differential expression of VIRMA and YTHDF3 among TGCT subtypes

In a preliminary analysis, no significant differences for *VIRMA* and *YTHDF3* transcript levels between pure (SE, EC and TE) and respective mixed tumor forms were found (Additional file 2: Figure S2). Thus, similar histological components derived from pure or MGCT were grouped together for subsequent analyses.

**Fig. 3:**
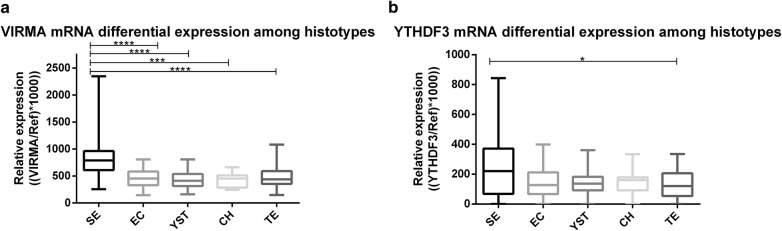
VIRMA (**a**) and YTHDF3 (**b**) mRNA expression levels among different tumor subtypes. *SE* Seminoma, *EC* embryonal carcinoma, *YST* pospubertal-type Yolk sac tumor, *CH* choriocarcinoma, *TE* postpubertal-type Teratoma, *Ref* reference genes GUSB and 18S

*VIRMA* transcript levels were significantly higher in SEs compared to all other NST components (adjusted *p* value: 0.0005 for SE *vs.* CH and < 0.0001 for SE *vs.* EC, YST or TE) (Fig. 3a). No significant differences were depicted among NST subtypes. As for *YTHDF3*, transcript levels were significantly higher in SEs compared to TEs, only (adjusted p-value 0.042) (Fig. 3b).

Considering all tumor samples, *VIRMA* and *YTHDF3* mRNA expression levels were positively correlated (r_s_ = 0.44, p < 0.001) (Additional file 3: Figure S3).

#### Association with clinicopathological parameters

**Fig. 4:**
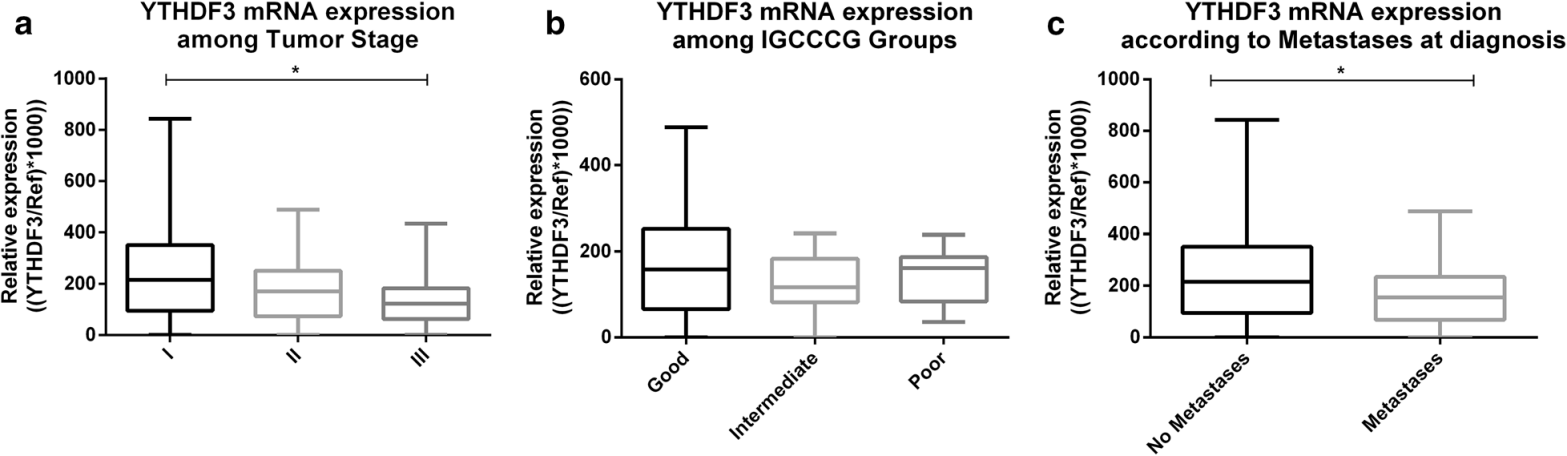
YTHDF3 transcript levels among Stage (**a**), IGCCCG Prognostic Group (**b**) and presence of metastases at diagnosis (**c**). IGCCCG international Germ Cell Cancer Collaborative Group; *Ref* reference genes GUSB and 18S

Significantly higher *YTHDF3* mRNA expression levels were observed in stage I compared to stage III TGCTs (adjusted p-value 0.0472), with a trend for decreasing expression in each disease stage. Also, patients with no metastases at diagnosis showed higher *YTHDF3* transcript levels compared to patients with metastases (p = 0.0212) (Fig. 4). Conversely, no significant associations between *VIRMA* expression levels and stage, IGCCCG grouping or metastatic disease status at diagnosis were depicted (Additional file 4: Figure S4).

### Evaluation of VIRMA, YTHDF3 and m^6^A immunoexpression

#### m^6^A, VIRMA and YTHDF3 immunoexpression in primary tumors

**Fig. 5:**
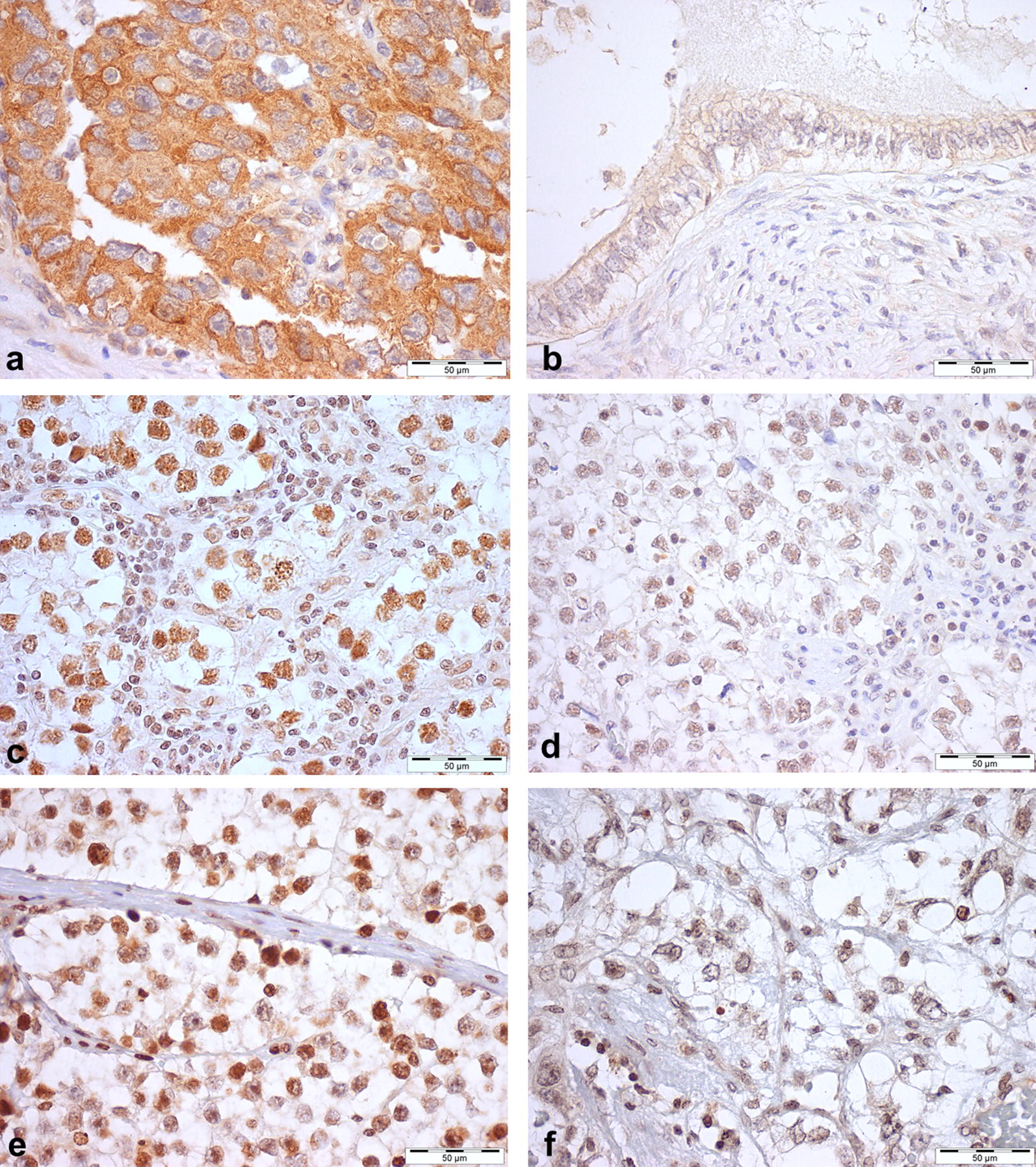
Immunostaining for YTHDF3 (**a**, **b**), VIRMA (**c**, **d**); and m^6^A (**e**, **f**) in testicular germ cell tumors. **a** Strong YTHDF3 cytoplasmic immunoexpression in embryonal carcinoma; **b** Weak/moderate YTHDF3 cytoplasmic immunoexpression in postpubertal-type teratoma; **c** strong VIRMA nuclear immunoexpression in seminoma; **d** Weak/moderate VIRMA nuclear immunoexpression in Seminoma; **e** Strong m^6^A nuclear immunostaining in seminoma; **f** Weak/moderate m^6^A nuclear immunostaining in postpubertal-type yolk sac tumor. Note the granularity of staining, particularly in **c**, **d** and **e**

The cellular distribution of the m^6^A modification, VIRMA and YTHDF3 differed among tested tumor tissues (detailed immunoexpression parameters are depicted in Additional file 5: Table S1). m^6^A immunostaining was predominantly nuclear [194/196 (99.0%) cases], with cytoplasmic staining in only 29.1% of tumor samples. Concerning m^6^A regulators, VIRMA staining was predominantly nuclear in all cases, whereas YTHDF3 exhibited mostly cytoplasmic staining. The nuclear staining of m^6^A and VIRMA and the cytoplasmic staining of YTHDF3 displayed a granular characteristic. Illustrative examples of immunostaining are depicted in Fig. 5.

**Fig. 6:**
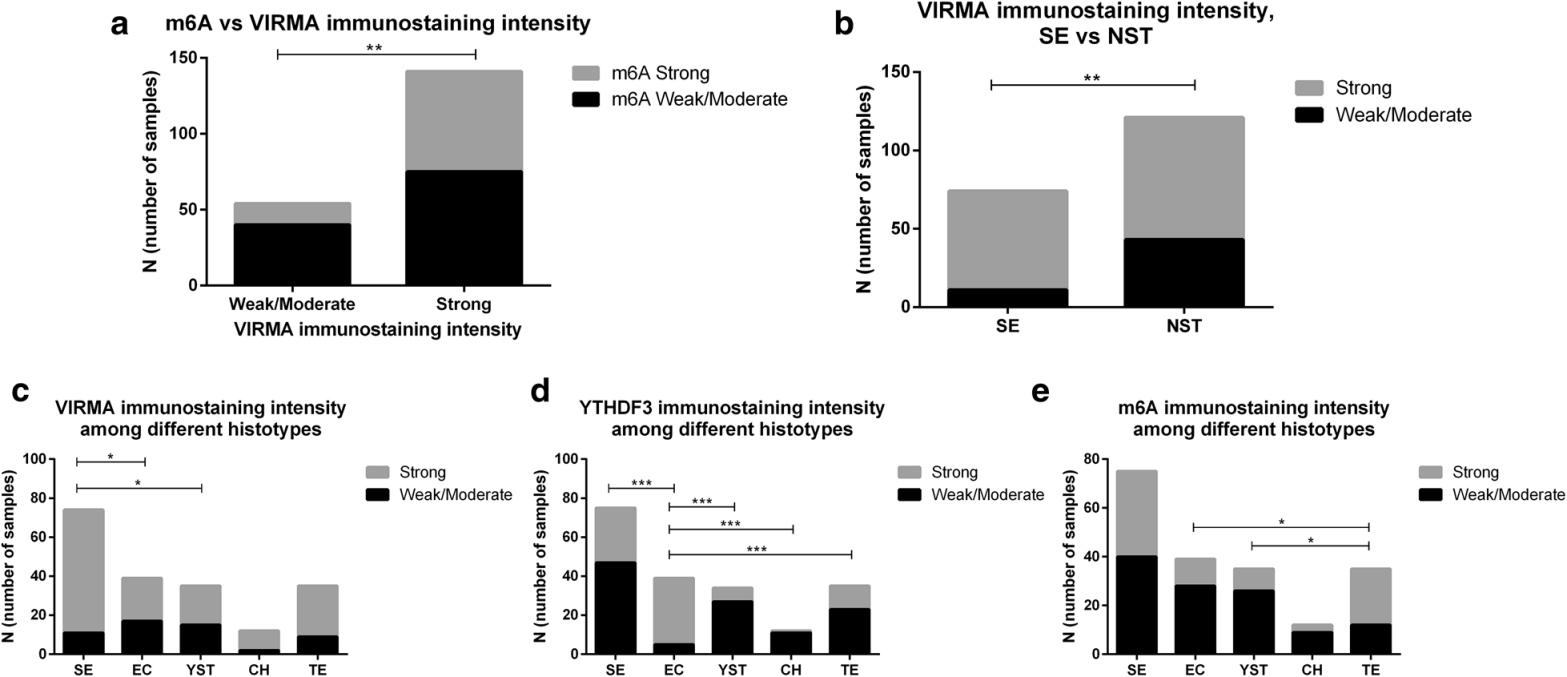
Immunostaining intensity of VIRMA, YTHDF3 and m^6^A. **a** m^6^A vs. VIRMA immunostaining intensity; **b** VIRMA immunostaining intensity among seminomas vs. non-seminomatous Tumors; **c**–**e** VIRMA, YTHDF3 and m^6^A immunostaining intensity among different tumor subtypes. *SE* Seminoma; *EC* embryonal carcinoma, *YST* pospubertal-type Yolk sac tumor, *CH* choriocarcinoma, *TE* postpubertal-type teratoma, *NST* non-seminomatous tumors

Immunostaining for m^6^A and VIRMA were significantly associated (p = 0.0092) (Fig. 6a). Indeed, the odds of having m^6^A strong immunoexpression was 2.5 times higher in samples with VIRMA strong staining intensity (OR = 2.514, 95% confidence interval [CI] 1.3–5.0). Contrarily, no significant association was found between m^6^A and YTHDF3 immunoexpression, neither between VIRMA and YTHDF3 immunoexpression.

Considering tumor types, a significant association between VIRMA immunoexpression and SE (p = 0.0017) was depicted (Fig. 6b). Indeed, SEs were 3 times more likely to exhibit VIRMA strong immunoexpression intensity (OR = 3.157, 95% CI 1.5–6.6). On the other hand, m^6^A and YTHDF3 immunoexpression intensity did not differ between SEs and NSTs as a whole.

Differential immunoexpression of VIRMA, YTHDF3 and m^6^A was observed among TGCT subtypes. SEs commonly displayed stronger VIRMA immunoexpression intensity compared to EC and YST (adjusted p-value 0.012 and 0.032, respectively), whereas ECs displayed the strongest YTHDF3 immunoexpression among all subtypes (adjusted p-value < 0.001 for all) and TEs showed stronger m^6^A immunoexpression than YSTs and ECs (adjusted p-value 0.016 and 0.022, respectively) (Fig. 6c–e).

#### m^6^A, VIRMA and YTHDF3 immunoexpression in metastases

A total of 19 metastatic specimens from 12 patients were available for immunoexpression analysis (tumor components in metastases that were also present in the orchiectomy specimen, Additional file 5: Table S1). Three and eight primary tumor samples with weak/moderate VIRMA and YTHDF3 immunoexpression, respectively, displayed strong immunoreactivity in the respective metastases, whereas disagreement in m^6^A immunoexpression between primary and metastatic samples occurred in four cases, all for from strong to weak/moderate (Additional file 6: Figure S5a–c).

Most metastatic tumor samples showed strong VIRMA and YTHDF3 immunoexpression intensity, while weak/moderate staining was observed for m^6^A (Additional file 6: Figure S5d–f).

Regarding TEs [primary tumors (n = 35) and metastases (n = 9)] strong YTHDF3 immunoexpression was more frequent in metastatic specimens (p = 0.0063), whereas m^6^A immunoexpression was stronger in primary tumors (p = 0.0270) (Additional file 6: Figure S5g–i).

### Association between immunoexpression and transcript levels

*VIRMA* transcript levels were significantly higher in tumor samples that exhibited strong VIRMA (p < 0.0001) and m^6^A (p = 0.0001) immunoexpression. No association was observed between VIRMA mRNA expression and YTHDF3 immunoexpression. Tumor samples with strong VIRMA immunoexpression also displayed significantly higher *YTHDF3* transcript levels (p = 0.0303) whereas those with strong YTHDF3 protein expression disclosed a trend for higher *YTHDF3* transcript levels (p = 0.0724) (Additional file 7: Figure S6).

## Discussion

Although relatively infrequent, TGCTs are highly curable, carrying a generally good prognosis. Nevertheless, and despite continuous improvement of multimodal treatments, reduced fertility and second neoplasms remain important side effects. On the other hand, 15–20% of patients with disseminated disease eventually relapse, entailing poor prognosis (especially late relapses) whereas others develop cisplatin resistance, by still elusive mechanisms, and the therapeutic strategy for these patients is suboptimal. Thus, novel disease biomarkers that may improve TGCT diagnosis and subtyping, prediction of disease progression and patient monitoring are needed, and their clinical implementation might aid in tailoring and individualizing therapeutic strategies [9, 11, 13, 46–52]. Over the past few years, more than 140 RNA modifications have been uncovered, as well as their context-dependent role in regulating target RNAs fate, namely in stability, translation and splicing [53]. Among these modifications, m^6^A seems, by far, the most abundant in mammalian mRNAs and a handful of enzymes that regulate this covalent modification have been characterized, including adenosine methyltransferases (writers), demethylating enzymes (erasers) and m^6^A-binding proteins (readers). Recently, the amount of m^6^A in RNAs, fine-tune regulated by those proteins, has been suggested to impact in cancer onset and progression. These players may also constitute therapeutic targets [19, 22, 24, 25, 28, 54], thus representing potential cancer biomarkers.

Considering the role of m^6^A modification in germ cell development, and being TGCTs considered development-related cancers [7, 55], we hypothesized that changes in abundance of this mark and/or altered expression of the enzymes involved in its writing, reading or erasing might be potential TGCT biomarkers. For selection of the best candidates among all m^6^A-related players, the TCGA dataset was surveyed, and *VIRMA* (a component of the m^6^A writer complex) and *YTHDF3* (reader) surfaced as the most commonly altered genes involved in m^6^A dynamics in TGCTs, prompting validation in an independent tissue cohort. Remarkably, SEs displayed higher *VIRMA* and *YTHDF3* transcript levels compared to NSTs, in both cohorts (TCGA’s and IPO Porto’s), notwithstanding some differences in composition. Indeed, although the proportion of SEs was similar (46% vs. 42%, in IPO Porto’s and TCGA cohorts, respectively), IPO Porto’s cohort globally displayed a lower proportion of stage I tumors (64% in IPO Porto’s vs. 79% in TCGA). Nonetheless, most metastatic tumors were within the Good IGCCCG Prognosis group in both series (71% and 74%, respectively). Moreover, median age at diagnosis was 28 years in our cohort and 31 years in TCGA’s, slightly lower than the 35 years reported in most studies, which might be due to the lower proportion of SEs in both series, as SEs tend to be diagnosed a decade later that NSTs [3, 4]. Thus, the overall characteristics of both cohorts might be considered globally representative of TGCT patients.

TGCTs tumorigenesis relates to biological processes of spermatogenesis and stem-cell differentiation [55], with SE constituting the so-called default pathway for GCNIS cells, whereas reprogramming originates NST components, including more undifferentiated and embryonal forms like EC, but also extra-embryonal forms like YST and CH, and more differentiated and somatic forms like TE [14, 51]. Because RNAs m^6^A chemical modification has been implicated in stemness state maintenance [37, 56–59], changes in abundance of m^6^A mark and differential expression of respective regulating proteins might be expected along differentiation of the various TGCT components. SEs displayed the highest *VIRMA* and *YTHDF3* mRNA levels, which were further confirmed at protein level for VIRMA, with no significant differences among NST subtypes. Considering their roles as m^6^A writer and reader, respectively, it is tempting to speculate whether m^6^A might contribute to the emergence and maintenance of the SE phenotype. Interestingly, knockdown of m^6^A writer complexes such as METTL3, METTL14, VIRMA, Hakai and WTAP lead to mouse embryonic stem cells (mESCs) self-renewal capacity loss, triggering differentiation [37, 56]. Hence, it has been proposed that m^6^A modification preferentially targets transcripts involved in development regulation, acting predominantly by reducing their stability and/or promoting degradation, maintaining mESCs at their ground state [37]. This observation is also in line with higher VIRMA expression observed in SEs compared to TEs, which represent the more differentiated TGCTs. TEs, however, showed the stronger m^6^A immunostaining intensity, suggesting that other writer complexes might be responsible for establishing m^6^A in this tumor subtype and/or that m^6^A modification might target other RNAs and even impart them a different fate. Intriguingly, this might parallel previous observations in mESCs, in which m^6^A displays conflicting results depending on the cell state: preserving stability of primed epiblast stem cells—EpiSCs—which are primed for differentiation, but inducing differentiation of naïve stem cells by degrading pluripotency factors [23, 58].

Overall, IHC results were concordant with transcript analysis, as tumors with strong VIRMA immunoexpression showed significantly higher VIRMA mRNA levels and a similar trend was depicted for YTHDF3. Moreover, a positive correlation between the writer and reader transcript levels was demonstrated in both cohorts, indicating they may cooperate in catalyzing and recognizing m^6^A modification in target RNAs. Furthermore, we found an association between higher expression of the writer (both at the transcript and immunohistochemical levels) and stronger m^6^A immunoexpression, suggesting that changes in m^6^A abundance might be due to VIRMA upregulation. Importantly, the observed immunoexpression patterns are coincidental with the expected subcellular localization. Indeed, VIRMA is mainly localized in nuclear bodies and nucleoplasm (correlating with granular/dot-like immunoexpression), whereas YTHDF3 is mostly cytoplasmic (especially in processing-bodies, imparting the granular staining observed in our samples) but it may also localize to nuclear speckles and nuclear membrane [19, 60–62]. Furthermore, m^6^A may be localized inside or outside the nucleus, imparting different biological significance [27], which may underlie the differences in immunoexpression depicted among distinct TGCT subtypes. On the other hand, immunostaining results in primary TGCTs and respective metastases was somewhat unexpected. Paradoxically, VIRMA and YTHDF3 immunostaining were increased in the metastatic context, whereas m^6^A modification displayed reduced intensity, suggesting that m^6^A erasure may occur during the metastatic process. In this context, a role for m^6^A erasers FTO and ALKBH5 in TGCT metastization is suggested. WTAP is fundamental for maintaining the writer complex inside the nuclear speckles, and it has been reported that WTAP knockdown results in m^6^A reduction mediated by sequestering of the writer complex in the cytoplasm; however, in our study, VIRMA immunoexpression in metastatic samples still occurred mainly in the nucleus [19, 63]. Nonetheless, our results should be interpreted with caution considering the limited number of metastatic samples and that most metastases were obtained following chemo- and/or radiotherapy, which may impact in m^6^A maintenance and detection.

Accurate discrimination of SEs from NST is key for patient management because SEs are particularly susceptible to chemo- and radiotherapy agents and display generally good prognosis, whereas NSTs show variable degrees of chemo- and radio-resistance and are more commonly associated with unfavorable prognostic features [6, 64, 65]. Thus, the ability of VIRMA transcript levels to discriminate SEs from NSTs is also of clinical significance and displayed similar results in both cohorts (AUC of 0.85 and 0.83 in IPO Porto’s and TCGA cohorts, respectively). Moreover, a gene panel comprising *VIRMA* and *YTHDF3* further increased sensitivity and specificity. If confirmed in liquid biopsies in the future, these results outperform those of classical TGCT serum markers [66, 67] and might constitute promising biomarkers for patient monitoring.

The retrospective nature of this study and the validation cohort size are important limitations of this study. Nevertheless, our cohort is similar in many respects to that of TCGA and reflects many aspects of TGCTs epidemiology. Furthermore, all patients were evaluated and treated in a single institution by the same multidisciplinary team, entailing homogeneity in patients’ staging and clinical management. Although m^6^A IHC assay is not ideal and qualitative analysis carries a considerable inter- and intra-observer variation, it is a widespread and accessible technique that may corroborate transcript findings.

## Conclusion

In summary, in this work we have used a bioinformatic tool to perform an in silico analysis, which allowed us to identify the most promising players to be further validated in a patient tissue cohort. We have shown that m^6^A writer VIRMA and reader YTHDF3 are differentially expressed among TGCTs subtypes, with significant overexpression in SEs compared to NSTs, suggesting a contribution to stemness maintenance. Furthermore, a positive correlation between VIRMA and YTHDF3 expression and an association with m^6^A abundance was also disclosed. Because VIRMA and YTHDF3 transcript levels accurately discriminate between SEs and NSTs, they might constitute novel biomarkers for patient management. Hence, our results confirm in silico findings and further enlighten the differentiation biology of TGCTs, which are development-related neoplasms. If confirmed in liquid biopsies, these players may also prove useful in the clinical setting.

To the best of our knowledge this is the first study assessing the value of m6A and related proteins in TGCTs. Additional research on m^6^A modification dynamics might further illuminate TGCTs biology and clinical behavior.

## Additional files

**Additional file 1: Figure S1.** In silico analysis: ROC curve for discrimination among seminomas and non-seminomatous tumors based on VIRMA mRNA expression levels using TCGA database. Abbreviations: SE: Seminoma; NST: non-seminomatous tumor; AUC: area under the curve; CI: confidence interval.

**Additional file 2: Figure S2.** Transcript levels of VIRMA and YTHDF3 among pure and matched mixed tumor forms. a VIRMA mRNA expression in pure seminoma vs. seminoma in mixed tumors; b VIRMA mRNA expression in pure embryonal carcinoma vs. embryonal carcinoma in mixed tumors; c VIRMA mRNA expression in pure teratoma vs. teratoma in mixed tumors; d YTHDF3 mRNA expression in pure seminoma vs. seminoma in mixed tumors; e YTHDF3 mRNA expression in pure embryonal carcinoma vs. embryonal carcinoma in Mixed tumors; f YTHDF3 mRNA expression in pure postpubertal-type teratoma vs. teratoma in mixed tumors. Abbreviations: SE: Seminoma; EC; embryonal carcinoma; TE: teratoma; Ref: reference genes GUSB and 18S.

**Additional file 3: Figure S3.** Correlation between mRNA expression levels of VIRMA and YTHDF3 in our cohort. Normalized for reference genes GUSB and 18S.

**Additional file 4: Figure S4.** VIRMA transcript levels among Stage (a), IGCCCG Prognostic Group (b) and presence of metastases at diagnosis (c). Abbreviations: IGCCCG: International Germ Cell Cancer Collaborative Group; Ref: reference genes GUSB and 18S.

**Additional file 5: Table S1.** Immunostaining for m6A, YTHDF3 and VIRMA in TGCT tumor samples.

**Additional file 6: Figure S5.** Immunohistochemistry findings in metastatic tumor samples. Comparison between immunostaining intensity of VIRMA (a), YTHDF3 (b) and m^6^A (c) between primary tumor samples and matched metastatic samples; immunostaining intensity of VIRMA (d), YTHDF3 (e) and m^6^A (f) among different tumor subtypes; comparison between immunostaining intensity of VIRMA (g), YTHDF3 (h) and m^6^A (i) in primary and metastatic Teratoma samples. Abbreviations: SE: Seminoma; EC: embryonal carcinoma; YST: pospubertal-type yolk sac tumor; CH: choriocarcinoma; Met: metastases; TE: postpubertal-type Teratoma.

**Additional file 7: Figure S6.** Association between transcript and immunohistochemistry findings. a VIRMA mRNA expression vs. VIRMA immunostaining; b VIRMA mRNA expression vs. YTHDF3 immunostaining; c VIRMA mRNA expression vs. m^6^A immunostaining; d YTHDF3 mRNA expression vs. YTHDF3 immunostaining; e YTHDF3 mRNA expression vs. VIRMA immunostaining; f YTHDF3 mRNA expression vs. m^6^A immunostaining.

## Abbreviations

AJCC: American Joint Committee on Cancer
AUC: area under the curve
cDNA: complementary DNA
CH: choriocarcinoma
DNA: deoxyribonucleic acid
EC: embryonal carcinoma
EpiSCs: epiblast stem cells
FFPE: formalin-fixed paraffin-embedded
GCNIS: germ cell neoplasia in situ
GUSB: beta-glucoronidase
IGCCCG: International Germ Cell Cancer Collaborative Group
IHC: immunohistochemistry
m6A: N-6-methyladenosine
mESCs: mouse embryonic stem cells
mRNA: messenger RNA
MGCT: mixed germ cell tumor
NPV: negative predictive value
NST: non-seminomatous tumor
OR: odds ratio
PPV: positive predictive value
RNA: ribonucleic acid
rRNA: ribosomal RNA
ROC: receiver operating characteristics
r_s_: Spearman’s correlation coefficient
RT-qPCR: real-time quantitative polymerase chain reaction
SE: seminoma
TCGA: The Cancer Genome Atlas
TE: postpubertal-type teratoma
TGCT: testicular germ cell tumor
WHO: World Health Organization
YST: postpubertal-type yolk sac tumor
