# Supplementary figures and images for: m6A RNA modification and its writer/reader VIRMA/YTHDF3 in testicular germ cell tumors: a role in seminoma phenotype maintenance
Source: J Transl Med. 2019 Mar 12;17:79. doi: 10.1186/s12967-019-1837-z (PMC6416960; doi:10.1186/s12967-019-1837-z)

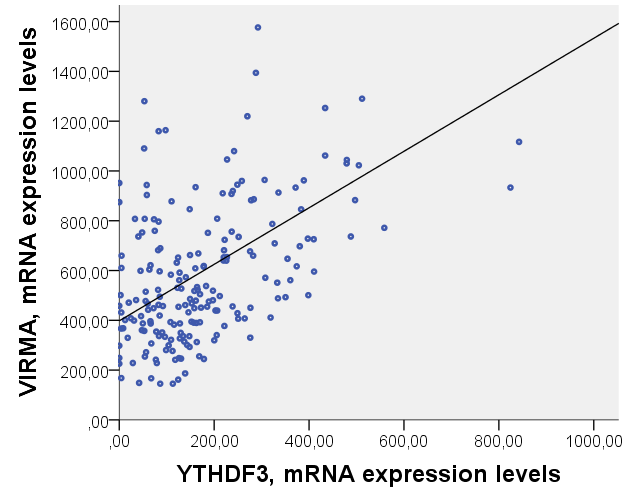

Supplement: Supplementary file 3 — Additional file 3: Figure S3. Correlation between mRNA expression levels of VIRMA and YTHDF3 in our cohort. Normalized for reference genes GUSB and 18S. [file 12967_2019_1837_MOESM3_ESM.tif]
