# Supplementary material for: m6A RNA modification and its writer/reader VIRMA/YTHDF3 in testicular germ cell tumors: a role in seminoma phenotype maintenance
Source: J Transl Med. 2019 Mar 12;17:79. doi: 10.1186/s12967-019-1837-z (PMC6416960; doi:10.1186/s12967-019-1837-z)
Supplement: Supplementary file 5 — Additional file 5: Table S1. Immunostaining for m6A, YTHDF3 and VIRMA in TGCT tumor samples. [file 12967_2019_1837_MOESM5_ESM.docx]

**Table S1 Immunostaining for m6A, YTHDF3 and VIRMA in TGCT tumor samples**

|  | m6A | YTHDF3 | VIRMA |
| --- | --- | --- | --- |
| Primary TGCT samples | | | |
| Intensity |  |  |  |
| Weak | 34/196 (17.4%) | 24/195 (12.3%) | 1/195 (0.5%) |
| Moderate | 81/196 (41.3%) | 89/195 (45.6%) | 53/195 (27.1%) |
| Strong | 81/196 (41.3%) | 82/195 (42.1%) | 141/195 (72.4%) |
| Compartment |  |  |  |
| Only Nuclear | 137/196 (69.9%) | 0/195 (0%) | 159/195 (81.5%) |
| Only Cytoplasmic | 2/196 (1.0%) | 148/195 (75.9%) | 0/195 (0%) |
| Nuclear and Cytoplasmic | 57/196 (29.1%) | 47/195 (24.1%) | 36/195 (18.5%) |
| Metastatic TGCT samples | | | |
| Intensity |  |  |  |
| Weak | 6/19 (31.6%) | 0/19 (0%) | 0/19 (0%) |
| Moderate | 8/19 (42.1%) | 3/19 (15.8%) | 3/19 (15.8%) |
| Strong | 5/19 (26.3%) | 16/19 (84.2%) | 16/19 (84.2%) |
| Compartment |  |  |  |
| Only Nuclear | 5/19 (26.3%) | 0/19 (0%) | 18/19 (94.7) |
| Only Cytoplasmic | 0/19 (0%) | 16/19 (84.2%) | 0/19 (0%) |
| Nuclear and Cytoplasmic | 14/19 (73.7%) | 3/19 (15.8%) | 1/19 (5.3%) |

**Abbreviations:** TGCT – Testicular germ cell tumor
